# Correlation of disease markers with clinical, endoscopic, and histologic findings in dogs with chronic gastrointestinal inflammation

**DOI:** 10.29374/2527-2179.bjvm001225

**Published:** 2025-09-11

**Authors:** Leda Marques de Oliveira-Barros, André Gustavo Alves Holanda, Romy Heilmann, Joerg Manfred Steiner, Genilson Fernandes de Queiroz, José Luiz Catão-Dias, Julia Maria Matera

**Affiliations:** 1 Departamento de Cirurgia, Faculdade de Medicina Veterinária e Zootecnia, Universidade de São Paulo (FMVZ/USP), São Paulo SP, Brazil; 2 Department of Small Animal Medicine, College of Veterinary Medicine University of Leipzig, Leipzig, Saxony, Germany; 3 Gastrointestinal Laboratory, College of Veterinary Medicine and Biomedical Sciences, Texas A&M University, College Station, Texas, United States; 4 Departamento de Patologia, Faculdade de Medicina Veterinária e Zootecnia, Universidade de São Paulo (FMVZ/USP), São Paulo SP, Brazil

**Keywords:** calgranulin, calprotectin, C-reactive protein, S100A12 protein, calgranulina, calprotectina, proteína C-reativa, proteína S100A12

## Abstract

The aim of this study was to evaluate serum and fecal biomarkers in dogs with chronic gastrointestinal inflammation and investigate their associations with clinical, endoscopic, and histopathological findings. Twenty dogs with chronic gastrointestinal signs and twenty clinically healthy control dogs were included. Dogs in the diseased group underwent clinical assessment, gastrointestinal endoscopy, and histopathological evaluation of intestinal biopsies. Serum concentrations of C-reactive protein (CRP), folate, cobalamin, calprotectin, and S100A12, as well as fecal concentrations of calprotectin and S100A12, were measured. Dogs with gastrointestinal inflammation showed significantly higher serum CRP and fecal calprotectin concentrations compared to healthy controls. Serum CRP was positively correlated with clinical disease activity scores and histologic severity in the colon. No significant differences in serum calprotectin or S100A12 concentrations were observed between groups. These findings suggest that serum CRP may serve as a supportive marker of disease severity and that fecal calprotectin holds promise as a non-invasive indicator of intestinal inflammation. In contrast, serum calprotectin and S100A12 showed limited diagnostic utility in this context. Overall, this study provides preliminary insights into the role of selected biomarkers in dogs with chronic gastrointestinal inflammation.

## Resumo

O objetivo deste estudo foi avaliar biomarcadores séricos e fecais em cães com inflamação gastrointestinal crônica e investigar suas associações com achados clínicos, endoscópicos e histopatológicos. Foram incluídos vinte cães com sinais gastrointestinais crônicos e vinte cães clinicamente saudáveis como grupo controle. Os cães do grupo doente foram submetidos à avaliação clínica, endoscopia gastrointestinal e análise histopatológica de biópsias intestinais. Foram mensuradas as concentrações séricas de proteína C reativa (PCR), folato, cobalamina, calprotectina e S100A12, bem como as concentrações fecais de calprotectina e S100A12. Cães com inflamação gastrointestinal apresentaram concentrações séricas de PCR e concentrações fecais de calprotectina significativamente mais altas em comparação aos controles saudáveis. A PCR sérica apresentou correlação positiva com os escores de atividade clínica da doença e com a gravidade histológica no cólon. Não foram observadas diferenças significativas nas concentrações séricas de calprotectina ou S100A12 entre os grupos. Esses achados sugerem que a PCR sérica pode atuar como um marcador de severidade da doença e que a calprotectina fecal apresenta potencial como um indicador não invasivo de inflamação intestinal. Em contraste, as concentrações séricas de calprotectina e S100A12 mostraram utilidade diagnóstica limitada nesse contexto. De modo geral, este estudo fornece evidências preliminares sobre o papel de biomarcadores selecionados na caracterização da gravidade da doença em cães com inflamação gastrointestinal crônica.

## Introduction

Chronic inflammatory enteropathy (CIE) in dogs is defined as chronic idiopathic inflammation (≥3 weeks) affecting one or more regions of the gastrointestinal tract. It is characterized by clinical signs such as vomiting, diarrhea, borborygmus, hyporexia, abdominal pain, nausea, and/or weight loss (Jergens & Heilmann, 2022). The diagnosis of CIE is established after the exclusion of extra-intestinal disorders, infectious or parasitic diseases, and other intestinal conditions, including mechanical obstructions caused by intussusception, foreign bodies, or intestinal tumors (Dandrieux, 2016).

Endoscopic biopsy of the gastrointestinal tract offers a rapid and relatively non-invasive method for direct assessment of mucosal lesions and allows targeted sampling of multiple gastrointestinal segments for histopathologic evaluation (Jergens & Heilmann, 2022). Histological examination of gastrointestinal biopsies confirm the presence of mucosal inflammation and to exclude diffuse neoplastic conditions and atypical infections (Dupouy-Manescau et al., 2024).

Treatment trials are often lengthy, and further diagnostic tests, such as endoscopy and histopathology, are more invasive than the use of biomarkers (Heilmann & Steiner, 2018). Biomarkers may aid in diagnostic evaluation, patient monitoring, or assessment of response to different forms of treatment, and therefore would be clinically useful in dogs with CIE by reflecting gastrointestinal inflammation and potentially reducing both the cost and time required for diagnosis (Sacoor et al., 2021). Several biomarkers, including serum albumin (Jablonski Wennogle et al., 2017), folate (Heilmann et al., 2018), cobalamin (Grützner et al., 2015), and C-reactive protein (CRP) (Grützner et al., 2015) and serum and fecal calprotectin (Heilmann et al., 2018) and S100A12 concentrations (Heilmann et al., 2014) have been proposed to evaluate disease activity in dogs with chronic enteropathy. However, these markers have not been studied extensively in dogs with CIE.

Albumin is a negative acute-phase protein, showing a trend towards reduced serum concentrations in the presence of systemic inflammatory disease processes that may reflect nutritional and inflammatory status (Allenspach, 2013; Wang et al., 2022). A serum albumin concentration of < 20 g/L is also an indicator of poor prognosis in canine CIE (Allenspach et al., 2007). Furthermore, serum concentrations of the vitamins cobalamin and folate in dogs with CIE differ from those in healthy animals, with hypocobalaminemia potentially serving as a prognostic indicator (Salavati Schmitz et al., 2019; Ullal et al., 2023).

CRP is a positive acute-phase protein. It may increase non-specifically due to inflammatory processes, including gastrointestinal diseases (Covin & Steiner, 2022). In dogs with CIE, serum CRP has also been associated with disease severity (Jergens et al., 2010) and response to treatment (Heilmann et al., 2018).

Calprotectin and S100A12 are proteins of the S100 family, released from activated mononuclear cells, and are classified as damage-associated molecular patterns (DAMPs). These proteins can be measured in both serum and fecal samples (Heilmann et al., 2019) and have been proposed as potential biomarkers for diagnosis and monitoring of treatment in dogs with CIE (Heilmann & Steiner, 2018).

We hypothesized that biomarkers may have diagnostic value and correlate with disease severity in dogs with chronic gastrointestinal inflammation (CGI). Therefore, this study aimed to evaluate serum and fecal biomarkers and their association with clinical, histopathological, and endoscopic severity scores in a population of dogs with CGI.

## Material and Methods

This study was approved by the Ethics Committee of the Faculty of Veterinary Medicine and Animal Science, University of São Paulo – FMVZ-USP (Protocol Nº1601/09).

### Animals

The sample included 20 clinically healthy client-owned dogs (control group) and 20 client-owned dogs with CGI of undetermined etiology (diseased group) referred to the Service of Small Animal Surgery, Surgery Department of FMVZ-USP. The owners were informed about the study before signing the informed consent form. The animals with gastrointestinal manifestations were prospectively evaluated and followed for a minimum period of three weeks.

The control group consisted of adult client-owned dogs with no history of gastrointestinal disorders. Health status was confirmed through history, physical examination, blood count, biochemical profile and fecal parasitology.

Dogs with CGI had clinical signs of chronic enteropathy (i.e., vomiting, diarrhea, and/or weight loss for ≥3 weeks) and other possible causes of chronic GI signs, including endoparasite infection, hepatic disease and renal disease were ruled out based on patient and vaccination history and physical and clinical examinations. Each dog underwent a general diagnostic evaluation, including complete blood cell counts, serum biochemical profiles, including measurement of the serum albumin concentration, reference interval [RI]: 23–38 g/L, fecal parasitology, and abdominal ultrasonography.

The animals were not subjected to dietary trials or deworming. No diet changes, antibiotic treatments, or immunosuppressant drugs were allowed in the four weeks preceding enrollment. There were no other restrictions to be included in the study. Specific diagnostic tests (e.g. trypsin-like immunoreactivity, resting cortisol) to rule out exocrine pancreatic insufficiency and hypoadrenocorticism were not performed.

### Clinical scoring

Six gastrointestinal clinical signs were semi-quantitatively assessed with the canine inflammatory bowel disease activity index (CIBDAI) (Jergens et al., 2003) prior to endoscopic examinations. These variables included attitude, appetite, vomiting, stool consistency, defecation frequency, and the presence of weight loss. Each parameter was graded based on severity as 0 (normal), 1 (mild), 2 (moderate), or 3 (severe). The sum of these individual scores defined the clinical disease activity score could range from 0 to 3 (interpreted as clinically insignificant disease), 4 to 5 (mild disease), 6 to 8 (moderate disease), or ≥9 (severe clinical illness). Although the animals in this study did not fully meet the established diagnostic criteria for chronic enteropathy, since comprehensive therapeutic trials were not performed, the CIBDAI scoring system was employed as a practical clinical tool to assess disease severity in dogs with CGI.

### Endoscopic scoring

Diseased dogs underwent esophagogastroduodenoscopy (EGD), colonoscopy, or EGD combined with colonoscopy, which were elected by the attending clinician according to the clinical manifestation of the disease. Endoscopic examinations were performed using a videogastroscope (GIF-H180, Olympus, Tokyo, Japan; external diameter: 9.8 mm, length: 108 cm, with a CV180 processor, Evis Exera II line, Olympus) and a high-quality light source (CLV180; Evis Exera II Line; Olympus). Endoscopic severity scores for the stomach, duodenum, and colon were established based on a scoring system (Allenspach et al., 2007). Scores of 0 (mucosa considered normal), 1 (mucosa with mild erythema, edema, and friable tissue), 2 (mucosa obviously edematous, erythematous, friable, and/or showing superficial and minute erosions), and 3 (mucosa with overt erythema, edema, and friable tissue showing easy bleeding, visible ulcerations, or both) were assigned. The maximum endoscopic score was determined by selecting the highest score among the gastrointestinal segments evaluated in an individual dog. All endoscopic procedures and scoring were performed by a single veterinary endoscopist

### Histopathological scoring

During endoscopy, at least three mucosal tissue biopsy samples were obtained using biopsy forceps (standard fenestrated model FB-25K-1, Olympus, and crocodile jaw model FB-220K.A, Olympus) from the body and antrum of the stomach, duodenum, and colon. Tissue samples were stored in 10% neutral-buffered formaldehyde, processed, and analyzed according to the classification proposed by the World Small Animal Veterinary Association Gastrointestinal Standardization group (Day et al., 2008). Each biopsy sample was classified according to the degree of alterations as 0 (normal tissue, absence of lesions), 1 (mild alterations), 2 (moderate alterations), or 3 (marked or severe lesions). These values were summed to determine the histologic lesion score for each patient in each anatomical segment evaluated. The maximum histopathological score was determined by selecting the highest score among the evaluated gastrointestinal segments in an individual dog.

### Serum and fecal biomarkers

Blood samples were obtained from diseased and control groups of dogs after a 12-hour fasting period to measure serum concentrations of CRP, calprotectin, S100A12, folate, and cobalamin. Blood samples from dogs with CGI were collected immediately before the endoscopy procedure. Fecal samples were collected over three consecutive days, which preceded the blood collection, to analyze calprotectin and S100A12 concentrations. All samples were stored frozen at -80°C until analysis. Serum and fecal calprotectin concentrations were measured using an in-house sandwich enzyme-linked immunosorbent assay (ELISA). A commercially available ELISA kit was used for serum CRP analysis (ELISA, Phase C Reactive Protein/CRP Canine kit, Tridelta, Maynooth, Ireland), which has been previously validated for use in dogs [RI: <7.6 mg/L;(Berghoff et al., 2006)]. A liquid-phase competitive radioimmunoassay (RIA) technique was used to measure S100A12 concentrations in serum and fecal samples [RI: 33–225 μg/L and <24–745 ng/g, respectively]. Serum folate and cobalamin concentrations [RI: 7.7–24.4 μg/L and 251-908 ng/L (Heilmann et al., 2017)] were determined using competitive solid-phase enzymatic chemiluminescence assays (Folic Acid-L2KFO6 and Vitamin B_12_-L2KVB6 kits, Siemens, Llanberis, Gwynedd, UK).

### Statistical analyses

Patient characteristics and clinical, endoscopic, and histopathological parameters were analyzed and reported using descriptive statistics. Categorical variables are represented by absolute and relative frequencies of occurrence, whereas continuous variables are reported as minimum, maximum, median, and/or mean values. Fecal concentrations of calprotectin and S100A12 for an individual dog were determined by averaging the two closest values and excluding the third most divergent result. Serum and/or fecal CRP, cobalamin, folate, calprotectin, and S100A12 concentrations were subjected to a Shapiro-Wilk test for normality testing. The concentrations of laboratory markers were non-normally distributed and compared between the diseased and control groups by using the Mann-Whitney *U* test. Correlations between biomarkers and clinical, endoscopic, and histopathological scores were evaluated by calculating Spearman's correlation coefficients (ρ). The correlation between biomarkers and endoscopic and histopathological scores was performed both by gastrointestinal segment and using the maximum score, which was determined by selecting the highest value among all evaluated segments. Statistical significance was set at a *p*-value of <0.05.

## Results

### Patient characteristic and clinical information

The median age of diseased dogs was two years (range: 1–10 years), and the median weight was 9.5 kg (range: 1.6–27 kg). Thirteen females (65%) and seven males (35%) were included in the diseased group. The most frequent clinical signs were vomiting (85%) and diarrhea (55%). Six dogs (30%) were considered to have clinically insignificant disease based on a CIBDAI score ≤3, 7 dogs (35%) had mild disease, 6 dogs (30%) had moderate disease, and one dog (5%) had severe disease. The average serum albumin concentration of the dogs included in the disease group was 32 g/L (range: 14–41 g/L). Hypoalbuminemia was detected in only 1 dog (5%). Details about the breed of dogs with CGI are shown in Table 1.

**Table 1:** Signalment and clinical characteristics of the dogs with chronic gastrointestinal inflammation (n=20) included in the study.

**Dog n^o^**	**Breed**	**Age (years)**	**Weight (kg)**	**Sex**	**Clinical Score** ^*^	**Clinical Signs**		
						**Vomiting**	**Diarrhea**	**Others**
**1**	Schnauzer	8	9.6	M	4	-	X	Dyschezia
**2**	Lhasa apso	4	4.5	F	6	X	-	-
**3**	Maltese	2	2.9	F	4	X	-	-
**4**	Yorkshire	2	3.7	F	2	X	-	-
**5**	Fox terrier	2	6.9	F	6	X	X	-
**6**	Poodle	1	6.8	F	5	-	X	Rectal prolapse
**7**	Mixed breed	5	1.6	M	6	X	-	Anorexia
**8**	Pitbull	7	27	M	8	-	X	Weight loss, hyporexia, dyschezia
**9**	Boxer	4	22.7	F	8	X	X	Weight loss, hyporexia, lethargy
**10**	Poodle	9	7	F	3	X	-	-
**11**	Mixed breed	10	8.5	F	0	X	-	Dysrexia
**12**	French bulldog	1	9.5	M	5	X	X	Rectal prolapse
**13**	Yorkshire terrier	1	3.5	F	5	X	X	Hyporexia
**14**	Boxer	1	24	M	3	X	X	-
**15**	Mixed breed	3	12	F	9	X	X	Weight loss, hyporexia
**16**	Whippet	7	8.8	F	3	X	-	Hyporexia
**17**	Lhasa apso	2	6.8	M	3	X	-	Hyporexia
**18**	Yorkshire terrier	2	3	F	5	X	X	-
**19**	Boxer	1	22.3	F	8	X	X	Weight loss, hyporexia, ascites
**20**	Yorkshire terrier	1	3	M	5	X	-	Hyporexia

* denotes the canine inflammatory bowel disease activity index (CIBDAI) score (Jergens et al. 2003).

F: female; M: male.

### Endoscopic evaluation

For the endoscopic evaluation, 9 dogs (45%) underwent an isolated EGD, 2 dogs (10%) an isolated colonoscopy, and 9 dogs (45%) an EGD combined with colonoscopy. Varying degrees of edema and erythema were observed in body and antrum of the stomach. Superficial erosions or deep gastric ulcerations were observed in four dogs (22%). In six (55%) of the 11 animals evaluated colonoscopically, it was not possible to introduce the endoscope through the ileocolic orifice. Four dogs (36%) had various degrees of edema and erythema in the colon, in addition to irregular, friable colonic mucosa with erosions or ulcerations. Seven dogs (64%) had erythema and erosions in the area of the ileocolic junction.

**Figure 1:**
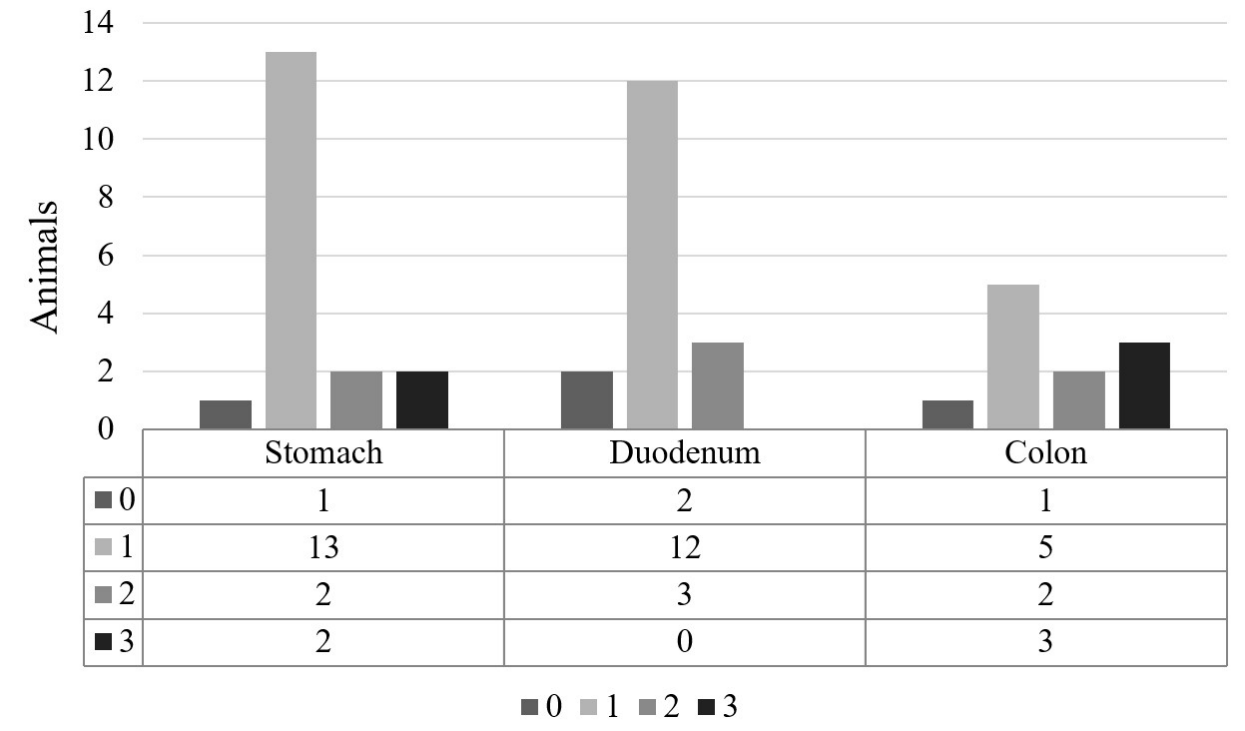
Distribution of endoscopic scores (0, 1, 2, 3) of stomach, duodenum and colon in dogs with chronic gastrointestinal inflammation.

Endoscopic scores could be determined for the stomach in 18 animals (100%), duodenum in 17 dogs (94%), and colon in 11 dogs (100%). In one animal, access to the duodenum was not possible due to the impossibility to introduce the endoscope through the pylorus, for anatomical reasons. Most dogs had a score of 1 for all compartments: 13 stomach (72%), 12 duodenum (70%), and 5 colon (45%). Therefore, the median endoscopic score was 1 for mucous membranes with the presence of mild erythema, edema, and tissue friability. Figure 1 summarizes the distribution of endoscopic scores in each gastrointestinal segment assessed in this study.

### Histopathological evaluation

Histopathological analyses were performed on the stomach of 18 dogs (100%), the duodenum of 15 dogs (88%), and the colon of 11 dogs (100%), all of which underwent endoscopic biopsy procedures. In two dogs (12%), duodenal histopathological analysis could not be performed due to sample loss during processing.

In relation to the stomach, evaluation of the mucosa in the gastric body revealed normal samples in 3 dogs (17%), lymphoplasmacytic inflammation in 12 dogs (67%), and non-inflammatory lesions (atrophy and fibrosis) in one dog (5%). Edema (30%), congestion (47%), and hemorrhage (12%) were also commonly observed. Two dogs (11%) had samples from only the gastric antrum evaluated, and the diagnosis for this segment was similar to that for the gastric body in both animals (moderate lymphoplasmacytic gastritis in one dog and severe lymphoplasmacytic gastritis in the other dog). Grading considered 9 parameters with a total of 162 assessments. Of these, 112 (69%) were considered normal, 42 (26%) mildly abnormal, and 6 (4%) as moderate alterations; two tissue samples (1%) could not be assigned grades because the representation of the tissue did not allow for a complete evaluation. The cumulative histology scores assigned for gastric lesions ranged from 0 to 9, with a median of 3.

Among the duodenal samples from 15 dogs, lymphoplasmacytic lesions were identified in 9 dogs (60%). Two dogs (13%) were diagnosed with neutrophilic enteritis, and another two (13%) with a mixed inflammatory infiltrate (i.e., containing eosinophils, lymphocytes, and neutrophils without the predominance of a single cell type). The tissue sample from one dog (7%) was classified as a non-inflammatory process (atrophy and fibrosis), and the sample from the remaining dog (7%) was considered normal. The evaluation included 10 parameters, totaling 150 assessments. Of these, 83 (55%) were categorized as normal, 54 (36%) as mild, 7 (5%) as moderate, and 2 (1%) as severe; 4 tissue samples (3%) could not be graded. The cumulative histology scores assigned to the duodenal samples ranged from 0 to 9, with a median of 5.

**Figure 2:**
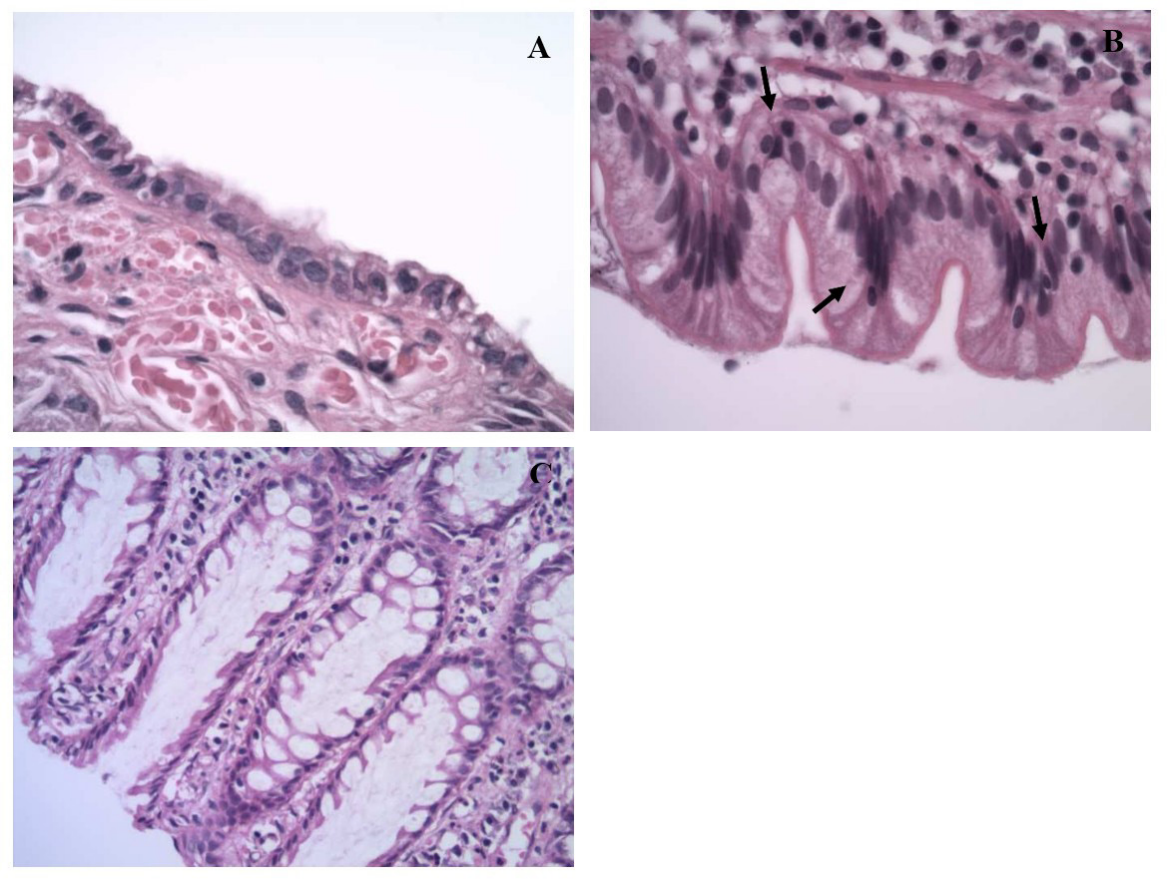
Photomicrographs of histological sections from dogs with chronic gastrointestinal inflammation. (A) Segment of the gastric body of dog nº 13 showing moderate epithelial injury (severity score of 2), flattening and degeneration of the columnar epithelium, HE, 400x magnification. (B) Duodenal segment of dog nº 2 demonstrating no epithelial injury to the villi but few intraepithelial lymphocytes as indicated by the arrows (severity score of 1), HE, 400x magnification. (C) Colonic segment of dog nº 5 with a normal crypt morphology and moderate numbers of eosinophils infiltrating the lamina propria, HE, 200x magnification.

Of the colonic samples evaluated from 11 dogs, nine (82%) revealed a lymphoplasmacytic process and one dog (9%) showed a predominantly eosinophilic inflammatory infiltrate. One dog (9%) exhibited a non-inflammatory process (showing atrophy and fibrosis). Evaluation included altogether 8 parameters, totaling 88 grades. Of these, 34 (39%) were assessed as normal, 40 (45%) as mild, and 14 (16%) as moderate. The cumulative histology scores for samples from the colon ranged from 3 to 10, with a median of 6. Figure 2 shows photomicrographs of exemplary histological sections from dogs with CGI.

### Biomarker concentrations

#### Serum C-reactive protein

Serum CRP concentrations in dogs with CGI (median: 1.9 mg/L, range: 0.1–44.0 mg/L) were significantly higher than those in healthy controls (median: 0.1 mg/L, range: 0.1–6.3 mg/L; *p*=0.028). Serum CRP concentrations were above the upper limit of the RI (>7.6 mg/L) in 5 (25%) dogs with CGI and in none of the healthy controls.

#### Serum and fecal calprotectin

Serum calprotectin concentrations in dogs with CGI (median: 8.8 mg/L, range: 2.4–42.2 mg/L) were not significantly different from those in healthy controls (median: 8.4 mg/L, range: 2.1–44.0 mg/L; *p*=0.779).

Fecal calprotectin concentrations differed from serum concentrations in that dogs with CGI (median: 0.4 μg/g, range: 0.4–165.1 μg/g) had significantly higher values than healthy controls (median: 0.4 μg/g, range: 0.4–35.0 μg/g; p = 0.026). Although the medians were the same, the broader range in the diseased group reflects greater variability and distribution of values, which contributed to the statistically significant difference.

#### Serum and fecal S100A12 protein

Serum S100A12 concentrations in dogs with CGI (median: 196 µg/L, range: 53–1,364 µg/L) were not significantly different from those in healthy controls (median: 196 µg/L, range: 88–1,010 µg/L; *p*=0.947). Serum S100A12 concentrations were above the upper limit of the RI in 9 (45%) dogs with CGI and in 8 (40%) healthy controls.

Fecal S100A12 protein concentrations also did not differ significantly between dogs with CGI (median: 25 ng/g, range: 3–5,411 ng/g) and those in the healthy control group (median: 12 ng/g, range: 3–2,160 ng/g; *p*=0.091). Fecal S100A12 protein concentrations were above the upper limit of the RI in 5 (25%) dogs with CGI and in 1 (5%) healthy control.

#### Serum folate and cobalamin

Serum folate concentrations showed no significant difference between dogs with CGI (median: 11.0 µg/L, range: 2.8–23.4 µg/L) and healthy controls (median: 12.9 µg/L, range: 1.4–28.2 µg/L; *p*=0.989). Serum folate concentrations were below the lower limit of the RI in 4 (20%) dogs with CGI and in 3 (15%) healthy controls. Hyperfolatemia was verified in only 1 (5%) healthy control.

Serum cobalamin concentrations were also similar between dogs with CGI (median: 491 ng/L, range: 255–1,000 ng/L) and the control group (median: 526 ng/L, range: 187–1,000 ng/L; *p*=0.640). Serum cobalamin concentrations were below the lower limit of the RI in none of the dogs with CGI and in 2 (10%) healthy controls. Hypercobalaminemia was verified in 1 (5%) dog of each group.

### Correlation among clinical variables and biomarker concentrations

There was a moderate positive correlation between serum CRP concentrations and clinical activity score (CIBDAI; ρ=0.52; *p*=0.019). Similarly, serum CRP concentrations correlated with the colonic histopathology score (ρ=0.633; *p*=0.037) and the maximum histologic lesions score (ρ=0.491; *p*=0.028). A strong positive correlation was observed between serum S100A12 and calprotectin concentrations (ρ=0.81; *p*=0.001) as well as between the respective fecal concentrations (ρ=0.94; *p*=0.001). Fecal calprotectin (ρ=-0.46; *p*=0.049) and fecal S100A12 concentrations were negatively correlated with serum albumin concentrations (ρ=-0.52; *p*=0.021).

## Discussion

The present study identified significant associations between selected biomarkers and disease features of CGI in dogs. Notably, serum CRP concentrations were significantly elevated in dogs with CGI and showed moderate positive correlations with clinical disease activity and histologic severity scores, suggesting its potential utility as a systemic marker of inflammation in this population. Additionally, while serum concentrations of calprotectin and S100A12 did not differ between groups, fecal calprotectin levels were significantly higher in diseased dogs, supporting its use as a non-invasive biomarker of intestinal inflammation.

Serum CRP concentrations were significantly higher in diseased dogs when compared to the control group, supporting the results of other studies (Jergens et al., 2003, 2010; Otoni et al., 2018; Tamura et al., 2019). Serum CRP concentrations were also positively correlated with the clinical disease activity index score, colonic histologic lesion score, and maximum histopathological score in the dogs with CGI. The inclusion of the maximum histopathological score was intended to reflect the most severe inflammatory lesion identified in any gastrointestinal segment of each individual. This approach was based on the heterogeneous and segmental distribution of lesions commonly observed in canine CIE (Jergens & Heilmann, 2022), where the most severely affected site may have the greatest influence on clinical signs and biomarker expression. However, the clinical value and reproducibility of this type of assessment require further investigation in future studies to determine its applicability and prognostic relevance.

Similar associations between serum CRP concentrations and clinical (Heilmann et al., 2018; Jergens et al., 2003, 2010) and histopathological (Heilmann et al., 2018; Jergens et al., 2003) severity scores have previously been reported. Thus, our study supports that serum CRP can serve as a surrogate marker to reflect disease severity in dogs with CGI. However, it needs to be emphasized that an increase in serum CRP concentrations is expected primarily in patients with moderate to severe disease (McCann et al., 2007). Furthermore, limitations for the clinical use of this biomarker in dogs include the high biological variability of serum concentrations (Carney et al., 2011) and the fact that several CRP assays are being used worldwide, making general conclusions difficult. In this study, the colon was the only intestinal segment for which the histologic lesion score correlated with serum CRP concentrations, likely because it had the lowest percentage of samples considered to be normal and the highest frequency of moderate to severe alterations. However, endoscopic and histological scores for the colon were available for only 11 animals, a notably small sample size that may significantly affect the robustness of the findings.

In our study, serum concentrations of the S100A12 protein were not significantly different between the healthy and diseased dogs. Differences in serum S100A12 concentrations were not observed between Chinese shar peis with and without hypocobalaminemia (Grützner et al., 2015). Other study described an increase in serum S100A12 concentrations in 15/18 dogs (83%) with protein-losing enteropathy and in 13/18 dogs (72%) with diet-responsive diarrhea, with no significant differences between the two groups (Equilino et al., 2015). In our study, evaluation of this inflammatory biomarker in fecal samples showed no significant difference between healthy and diseased dogs. Increased fecal S100A12 concentrations in dogs with CIE have been previously reported. However, the specificity and sensitivity of this test with an established cut-off point of 59 ng/g were only 84% and 65%, respectively (Heilmann et al., 2014).

The expression of S100A12 in the gastrointestinal tract has been primarily associated with inflammatory cell infiltrates composed of neutrophils and, to a lesser extent, macrophages (Heilmann et al., 2019). In contrast, inflammatory infiltrates in dogs with CIE are typically dominated by lymphocytes, plasma cells, and eosinophils, with neutrophils generally underrepresented (Day et al., 2008). This cellular profile may partially explain the limited variation in S100A12 concentrations observed in most affected dogs. In our study, both dogs that exhibited neutrophilic infiltration showed markedly increased fecal S100A12 concentrations (1,446 ng/g and 3,727 ng/g), and one of them also had an elevated serum S100A12 level (381 μg/L). However, serum concentrations of this protein are not specific to gastrointestinal disease and, similar to CRP, may also be elevated in other systemic inflammatory conditions, including pancreatitis (Jandel et al., 2023), sepsis (Thames et al., 2019), and hyperlipidemia (Heilmann et al., 2019).

Fecal calprotectin concentrations were significantly higher in diseased dogs than in healthy controls, which is in line with findings from a previous investigation (Otoni et al., 2018). In contrast, no significant differences in serum calprotectin concentrations were observed between the groups in the present study. Significant differences in serum calprotectin concentrations between dogs with CIE and healthy controls have been reported; however, the sensitivity and specificity of the RIA assay with an established cutoff point of 296 μg/L were only 82% and 68%, respectively (Heilmann et al., 2012).

Associations between fecal calprotectin concentrations and the clinical disease activity score have been reported in dogs with CIE (Heilmann et al., 2018; Otoni et al., 2018). Similarly, an association was documented for fecal S100A12 concentrations with clinical severity scores, colonic histologic lesion scores, and endoscopic scores for the duodenum and colon (Heilmann et al., 2014). These results are in contrast with our findings as we were unable to verify such correlations of fecal calprotectin and/or S100A12 concentrations with clinical, endoscopic, and/or histological scores. However, such an association may have been obscured due to the small number of dogs evaluated and the large percentage of dogs with mild disease severities included in the current study.

We detected a correlation between the fecal concentrations of S100A12 and calprotectin, and also between their serum concentrations. Associations of these proteins in the blood suggest they are derived from circulating leukocytes and may reflect systemic rather than localized intestinal inflammatory responses (Fukunaga et al., 2017). Thus, it would be expected that the measurement of these inflammatory biomarkers in fecal specimens is more specific to evaluate the intestinal tract in dogs with CGI. However, unlike calprotectin, fecal S100A12 concentrations were not significantly different between healthy and diseased dogs.

Although only one dog in our study was hypoalbuminemic, serum albumin concentrations were negatively correlated with both fecal calprotectin and S100A12 concentrations. While this finding may reflect an association between intestinal inflammation and albumin status, it must be interpreted with caution due to the limited number of hypoalbuminemic animals. Therefore, this observation is exploratory and highlights the need for further studies to assess whether fecal S100 family proteins may correlate with disease severity or prognostic indicators such as hypoalbuminemia in dogs with CGI.

Serum cobalamin (vitamin B_12_) measurement, often in conjunction with that of serum folate, is routinely performed in small animal clinical practice to diagnose B vitamin deficiencies (Kather et al., 2020). Changes in these markers in patients with chronic intestinal disease have been suggested to be associated with disease location (Simpson & Jergens, 2011) as cobalamin is absorbed primarily within the ileum, whereas folate is absorbed more proximally in the duodenum and proximal jejunum (Heilmann & Steiner, 2018). In this study, serum cobalamin and folate concentrations did not differ between the two groups of dogs, but reduced serum cobalamin concentrations have previously been observed in dogs with chronic enteropathies, and hypocobalaminemia is generally associated with a poorer prognosis (Allenspach et al., 2007; Berghoff et al., 2013; Volkmann et al., 2017). Malabsorption could be an important factor related to cobalamin and folate deficiencies. However, as opposed to the previous theory, increased receptor expression levels were recently confirmed in hypocobalaminemic dogs with CIE (Kather et al., 2024). Hence, some dogs may experience a higher demand for these vitamins or the presence of small intestinal dysbiosis (SID) associated with an increased folate synthesis and reduced cobalamin uptake due to bacterial utilization (Dossin, 2011; Heilmann & Steiner, 2018). In this study, the mild severity of the lesions and the dietary effects (including the possibility of prior supplementation) were likely the main reasons for the lack of differences between the groups.

This study has several limitations. The dogs were not tested to rule out pancreatitis (pancreatic lipase immunoreactivity), exocrine pancreatic insufficiency (canine trypsin-like immunoreactivity), or hypoadrenocorticism (resting cortisol or adrenocorticotropic hormone stimulation test), all of which can cause gastrointestinal signs. Therefore, we cannot definitively conclude that the gastrointestinal inflammation observed was attributable to CIE. In addition, cholesterol and triglyceride levels were not measured, which could potentially influence the interpretation of inflammatory biomarkers such as CRP and S100A12. The study population was small, and a large proportion of dogs had only mild disease, which may have limited our ability to detect significant associations. We cannot exclude the possibility that these factors contributed to type II statistical errors.

In conclusion, serum CRP concentrations were correlated with indices of disease severity and may serve as a supportive marker in the assessment of dogs with CGI and suspected chronic enteropathy. In contrast, serum calprotectin and S100A12 concentrations did not differ significantly between healthy and diseased dogs, limiting their diagnostic utility in this context. Fecal calprotectin concentrations were significantly higher in diseased dogs, suggesting its potential as a non-invasive indicator of intestinal inflammation. These findings provide preliminary insights into the role of selected biomarkers in characterizing disease severity in dogs with CGI.

## References

[B001] Allenspach K. (2013). Diagnosis of small intestinal disorders in dogs and cats. The Veterinary Clinics of North America. Small Animal Practice.

[B002] Allenspach K., Wieland B., Gröne A., Gaschen F. (2007). Chronic enteropathies in dogs: Evaluation of risk factors for negative outcome. Journal of Veterinary Internal Medicine.

[B003] Berghoff N., Parnell N. K., Hill S. L., Suchodolski J. S., Steiner J. M. (2013). Serum cobalamin and methylmalonic acid concentrations in dogs with chronic gastrointestinal disease. American Journal of Veterinary Research.

[B004] Berghoff N., Suchodolski J. S., Steiner J. M. (2006). Assessment of stability and determination of a reference range for canine c-reactive protein in serum. In: Research Abstract Program of the 24th Annual ACVIM Forum Louisville, KY, May 31 ‐ June 3, 2006. Journal of Veterinary Internal Medicine.

[B005] Carney P. C., Ruaux C. G., Suchodolski J. S., Steiner J. M. (2011). Biological variability of C‐Reactive protein and specific canine pancreatic lipase immunoreactivity in apparently healthy dogs. Journal of Veterinary Internal Medicine.

[B006] Covin M. A., Steiner J. M. (2022). Measurement and clinical applications of C‐reactive protein in gastrointestinal diseases of dogs. Veterinary Clinical Pathology.

[B007] Dandrieux J. R. S. (2016). Inflammatory bowel disease versus chronic enteropathy in dogs: Are they one and the same?. The Journal of Small Animal Practice.

[B008] Day M. J., Bilzer T., Mansell J., Wilcock B., Hall E. J., Jergens A., Minami T., Willard M., Washabau R., World Small Animal Veterinary Association Gastrointestinal Standardization Group (2008). Histopathological Standards for the Diagnosis of Gastrointestinal Inflammation in Endoscopic Biopsy Samples from the Dog and Cat: A Report from the World Small Animal Veterinary Association Gastrointestinal Standardization Group. Journal of Comparative Pathology.

[B009] Dossin O. (2011). Laboratory tests for diagnosis of gastrointestinal and pancreatic diseases. Topics in Companion Animal Medicine.

[B010] Dupouy-Manescau N., Méric T., Sénécat O., Drut A., Valentin S., Leal R. O., Hernandez J. (2024). Updating the classification of chronic inflammatory enteropathies in dogs. Animals (Basel).

[B011] Equilino M., Théodoloz V., Gorgas D., Doherr M. G., Heilmann R. M., Suchodolski J. S., Steiner J. M., Burgener D. V. M. (2015). Evaluation of serum biochemical marker concentrations and survival time in dogs with protein-losing enteropathy. Journal of the American Veterinary Medical Association.

[B012] Fukunaga S., Kuwaki K., Mitsuyama K., Takedatsu H., Yoshioka S., Yamasaki H., Yamauchi R., Mori A., Kakuma T., Tsuruta O., Torimura T. (2017). Detection of calprotectin in inflammatory bowel disease: Fecal and serum levels and immunohistochemical localization. International Journal of Molecular Medicine.

[B013] Grützner N., Heilmann R. M., Cranford S. M., Holzenburg A., Suchodolski J. S., Steiner J. M. (2015). Inflammatory, immunological, and intestinal disease biomarkers in Chinese Shar-Pei dogs with marked hypocobalaminemia. Journal of Veterinary Diagnostic Investigation.

[B014] Heilmann R. M., Steiner J. M. (2018). Clinical utility of currently available biomarkers in inflammatory enteropathies of dogs. Journal of Veterinary Internal Medicine.

[B015] Heilmann R. M., Berghoff N., Mansell J., Grützner N., Parnell N. K., Gurtner C., Suchodolski J. S., Steiner J. M. (2018). Association of fecal calprotectin concentrations with disease severity, response to treatment, and other biomarkers in dogs with chronic inflammatory enteropathies. Journal of Veterinary Internal Medicine.

[B016] Heilmann R. M., Grellet A., Allenspach K., Lecoindre P., Day M. J., Priestnall S. L., Toresson L., Procoli F., Grützner N., Suchodolski J. S., Steiner J. M. (2014). Association between fecal S100A12 concentration and histologic, endoscopic, and clinical disease severity in dogs with idiopathic inflammatory bowel disease. Veterinary Immunology and Immunopathology.

[B017] Heilmann R. M., Grützner N., Iazbik M. C., Lopes R., Bridges C. S., Suchodolski J. S., Couto C. G., Steiner J. M. (2017). Hyperhomocysteinemia in greyhounds and its association with hypofolatemia and other clinicopathologic variables. Journal of Veterinary Internal Medicine.

[B018] Heilmann R. M., Jergens A. E., Ackermann M. R., Barr J. W., Suchodolski J. S., Steiner J. M. (2012). Serum calprotectin concentrations in dogs with idiopathic inflammatory bowel disease. American Journal of Veterinary Research.

[B019] Heilmann R. M., Nestler J., Schwarz J., Grützner N., Ambrus A., Seeger J., Suchodolski J. S., Steiner J. M., Gurtner C. (2019). Mucosal expression of S100A12 (calgranulin C) and S100A8/A9 (calprotectin) and correlation with serum and fecal concentrations in dogs with chronic inflammatory enteropathy. Veterinary Immunology and Immunopathology.

[B020] Jablonski Wennogle S. A., Priestnall S. L., Webb C. B. (2017). Histopathologic Characteristics of Intestinal Biopsy Samples from Dogs With Chronic Inflammatory Enteropathy With and Without Hypoalbuminemia. Journal of Veterinary Internal Medicine.

[B021] Jandel A. N., Heilmann R. M., Sander H., Steiner J. M., Grützner N., Xenoulis P. G. (2023). Serum α1-Proteinase inhibitor, calprotectin, and S100A12 concentrations in the characterization of pancreatitis in dogs. Veterinary Sciences.

[B022] Jergens A. E., Heilmann R. M. (2022). Canine chronic enteropathy: Current state-of-the-art and emerging concepts. Frontiers in Veterinary Science.

[B023] Jergens A. E., Crandell J., Morrison J. A., Deitz K., Pressel M., Ackermann M., Suchodolski J. S., Steiner J. M., Evans R. (2010). Comparison of oral prednisone and prednisone combined with metronidazole for induction therapy of canine inflammatory bowel disease: A randomized-controlled trial. Journal of Veterinary Internal Medicine.

[B024] Jergens A. E., Schreiner C. A., Frank D. E., Niyo Y., Ahrens F. E., Eckersall P. D., Benson T. J., Evans R. (2003). A scoring index for disease activity in canine inflammatory bowel disease. Journal of Veterinary Internal Medicine.

[B025] Kather S., Grützner N., Kook P. H., Dengler F., Heilmann R. M. (2020). Review of cobalamin status and disorders of cobalamin metabolism in dogs. Journal of Veterinary Internal Medicine.

[B026] Kather S., Kacza J., Pfannkuche H., Böttcher D., Sung C.-H., Steiner J. M., Gäbel G., Dengler F., Heilmann R. M. (2024). Expression of the cobalamin transporters cubam and MRP1 in the canine ileum–Upregulation in chronic inflammatory enteropathy. PLoS One.

[B027] McCann T. M., Ridyard A. E., Else R. W., Simpson J. W. (2007). Evaluation of disease activity markers in dogs with idiopathic inflammatory bowel disease. The Journal of Small Animal Practice.

[B028] Otoni C. C., Heilmann R. M., García‐Sancho M., Sainz A., Ackermann M. R., Suchodolski J. S., Steiner J. M., Jergens A. E. (2018). Serologic and fecal markers to predict response to induction therapy in dogs with idiopathic inflammatory bowel disease. Journal of Veterinary Internal Medicine.

[B029] Sacoor C., Barros L. M., Montezinho L. (2021). What are the potential biomarkers that should be considered in diagnosing and managing canine chronic inflammatory enteropathies?. Open Veterinary Journal.

[B030] Salavati Schmitz S., Gow A., Bommer N., Morrison L., Mellanby R. (2019). Diagnostic features, treatment, and outcome of dogs with inflammatory protein‐losing enteropathy. Journal of Veterinary Internal Medicine.

[B031] Simpson K. W., Jergens A. E. (2011). Pitfalls and Progress in the Diagnosis and Management of Canine Inflammatory Bowel Disease. The Veterinary Clinics of North America. Small Animal Practice.

[B032] Tamura Y., Ohta H., Kagawa Y., Osuga T., Morishita K., Sasaki N., Takiguchi M. (2019). Plasma amino acid profiles in dogs with inflammatory bowel disease. Journal of Veterinary Internal Medicine.

[B033] Thames B. E., Barr J. W., Suchodolski J. S., Steiner J. M., Heilmann R. M. (2019). Prospective evaluation of S100A12 and S100A8/A9 (calprotectin) in dogs with sepsis or the systemic inflammatory response syndrome. Journal of Veterinary Diagnostic Investigation.

[B034] Ullal T. V., Marks S. L., Huebner S. N., Taylor S. L., Shelley C. D. (2023). Association of folate concentrations with clinical signs and laboratory markers of chronic enteropathy in dogs. Journal of Veterinary Internal Medicine.

[B035] Volkmann M., Steiner J. M., Fosgate G. T., Zentek J., Hartmann S., Kohn B. (2017). Chronic diarrhea in dogs – retrospective study in 136 cases. Journal of Veterinary Internal Medicine.

[B036] Wang Y., Li C., Wang W., Wang J., Li J., Qian S., Cai C., Liu Y. (2022). Serum albumin to globulin ratio is associated with the presence and severity of inflammatory bowel disease. Journal of Inflammation Research.

